# Supplementary material for: A machine learning toolbox for the analysis of sharp-wave ripples reveals common waveform features across species
Source: Commun Biol. 2024 Mar 4;7:211. doi: 10.1038/s42003-024-05871-w (PMC10912113; doi:10.1038/s42003-024-05871-w)
Supplement: Supplementary file 1 — Supplementary material [file 42003_2024_5871_MOESM1_ESM.pdf]

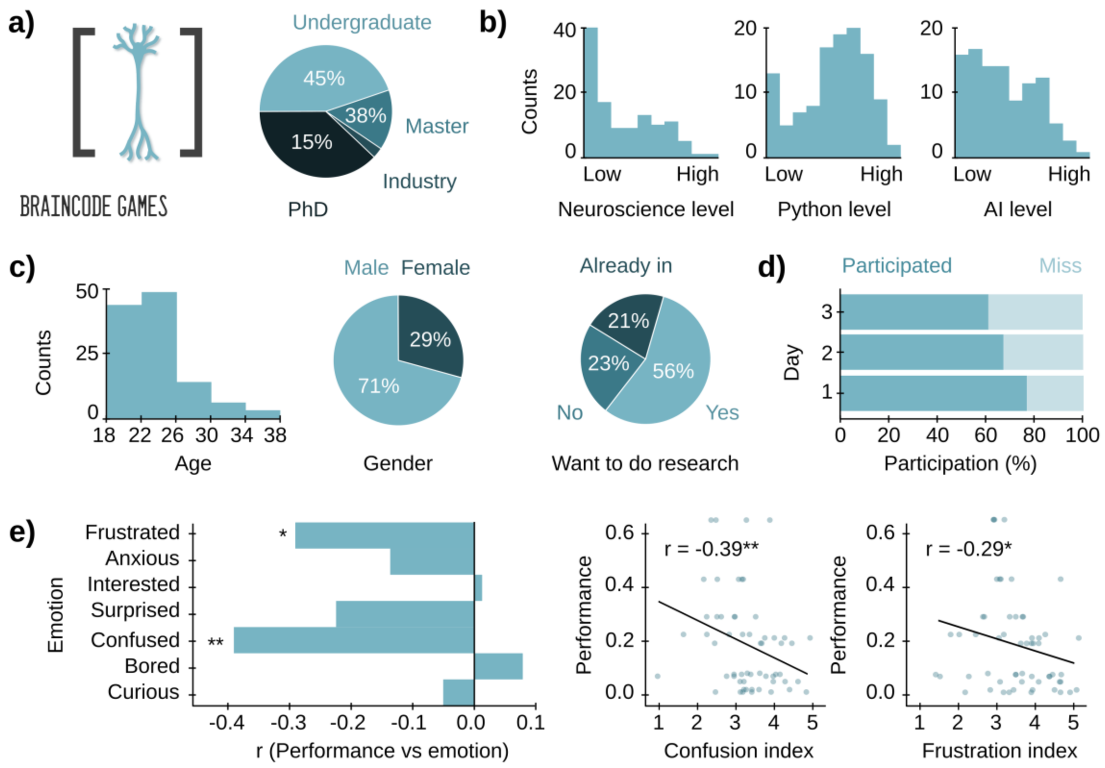

Supplementary Figure 1. Information about the hackathon. a). A hackathon was organized to seek for community-based solutions to the SWR challenge from people unfamiliar to SWR neurophysiology. Among the 116 participants, there were undergraduate students (45%), master students (38%), PhD students (15%), and industry workers (3%). **b)** There was a general lack of neuroscience knowledge, although most participants declared a high-level performance in Python. Most groups integrated people with programming abilities and basic ML knowledge. **c)** Participant age (left), gender (middle; 71% male, 29% female participants) and involvement in research (right; 21% already in research; 56% interested in doing basic research; 23% not motivated for basic research activities). **d)** Self-reported participation rate during the three days of the hackathon. **e)** Correlation between the performance metric of the proposed solution and emotional states of participants as quantified from their responses to surveys recorded during the hackathon (Spearman rank-order correlation *, $p<0.05$; **, $p<0.01$). Only performance of functional solutions was used. See Methods for details.

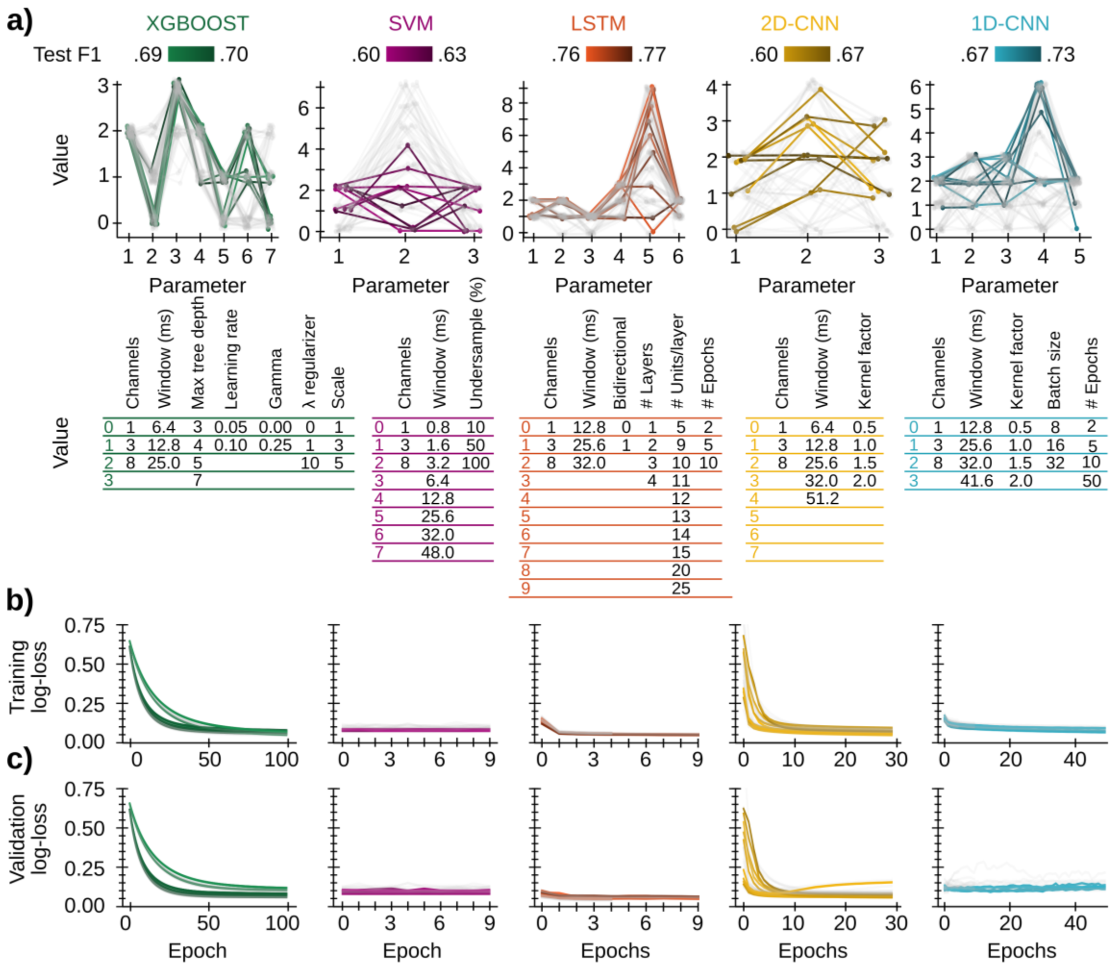

XGBOOST

Value	Channels	Window (ms)	Max tree depth	Learning rate	Gamma	λ regularizer	Scale
0	1	6.4	3	0.05	0.00	0	1
1	3	12.8	4	0.10	0.25	1	3
2	8	25.0	5			10	5
3			7				

SVM

Value	Channels	Window (ms)	Undersample (%)
0	1	0.8	10
1	3	1.6	50
2	8	3.2	100
3		6.4	
4		12.8	
5		25.6	
6		32.0	
7		48.0	

LSTM

Value	Channels	Window (ms)	Bidirectional	# Layers	# Units/layer	# Epochs
0	1	12.8	0	1	5	2
1	3	25.6	1	2	9	5
2	8	32.0		3	10	10
3				4	11	
4					12	
5					13	
6					14	
7					15	
8					20	
9					25	

2D-CNN

Value	Channels	Window (ms)	Kernel factor
0	1	6.4	0.5
1	3	12.8	1.0
2	8	25.6	1.5
3		32.0	2.0
4		51.2	
5			
6			
7			

1D-CNN

Value	Channels	Window (ms)	Kernel factor	Batch size	# Epochs
0	1	12.8	0.5	8	2
1	3	25.6	1.0	16	5
2	8	32.0	1.5	32	10
3		41.6	2.0		50

Supplementary Figure 2. Definition of parameter space in the different ML architectures. a) Results from the different architectures in the training dataset: XGBoost, SVM, LSTM, 2D-CNN and 1D-CNN. Tables indicate the different hyper-parameters used to train each architecture. The resulting 10-best models are color-coded by their F1-score in the test dataset. The remaining 40-best models are shown in light gray. **b)** Evolution of logarithmic-loss (a metric for error prediction that takes into account unbalanced data) along training epochs using the training dataset for the ML models shown in A. **c)** Same as b), but using the validation dataset.

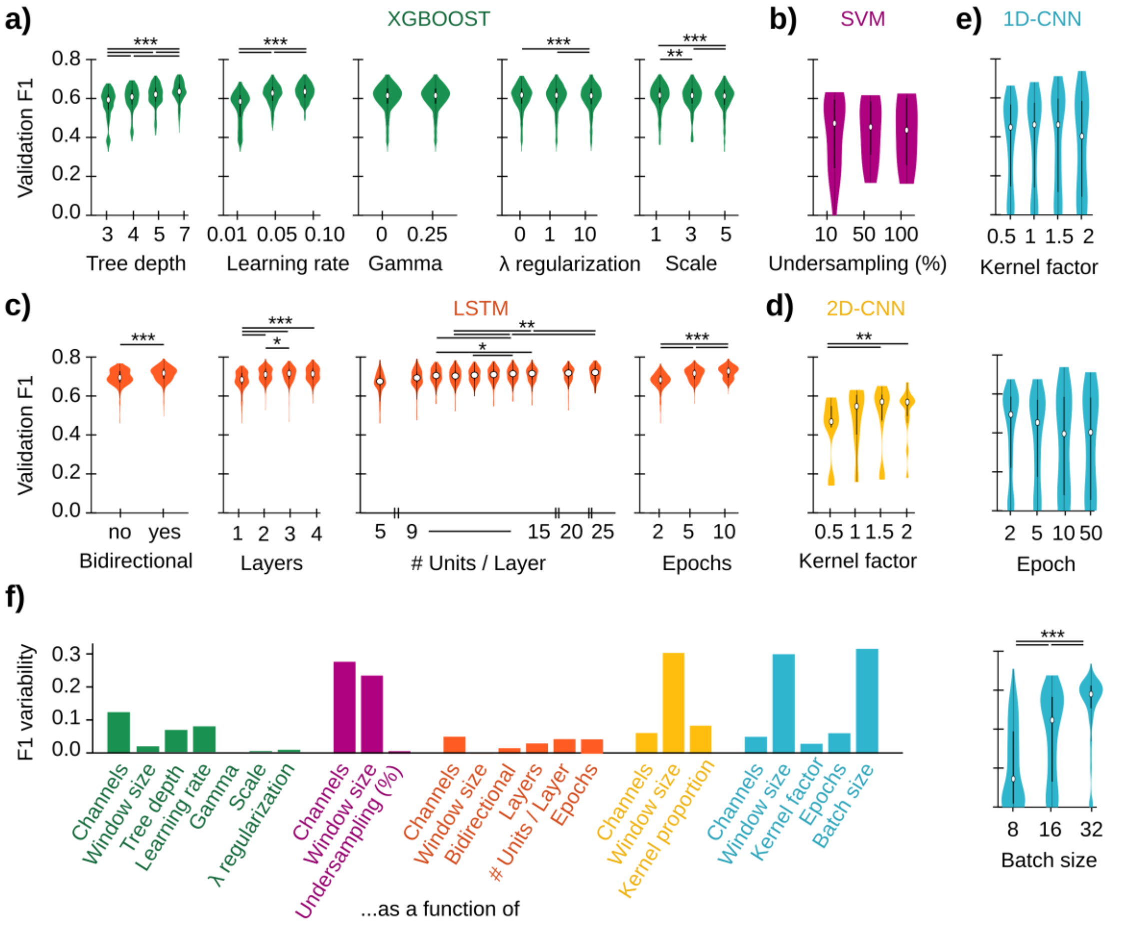

Supplementary Figure 3. Influence of architecture-specific training parameters on performance. a) to e), F1-scores from the validation set for all models of each architecture. All statistical tests were Kruskal Wallis (KW) with repeated measures. **a)** XGBoost training parameters: maximum tree depth (KW: Chi2(3)=1321.6, p<0.0001), learning rate (KW: Chi2(2)=1109.4.6, p<0.0001), gamma (KW not significant), lambda regularization (KW: Chi2(2)=67.8, p<0.0001) and scale (KW: Chi2(2)=111.6, p<0.0001). Post hoc tests: *, p<0.05; **, p<0.01, ***, p<0.001. **b)** SVM training parameters: under-sampling percentage (KW not significant). Higher % of undersampling means training the model with higher representativity of GT data. **c)** LSTM training parameters: bidirectionality (KW: Chi2(1)=320.1, p<0.0001), number of layers KW: Chi2(3)=602.4, p<0.0001), number of units per layer (KW: Chi2(9)=543.8, p<0.0001) and training epochs (KW: Chi2(2)=836.1.6, p<0.0001). **d)** 2D-CNN training parameters: number of kernels scaling factor (KW: Chi2(3)=16.0, p=0.0011), number of epochs and batch size (KW not significant). **e)** 1D-CNN training parameters: number of kernels scaling factor (KW not significant), number of training epochs (KW not significant), and batch size (KW: Chi2(2)=196.9, p<0.0001). **f)** F1-score variability as a function of all training parameters. F1 variability was computed as the difference between the maximum and minimum mean F1. For a-e graphs, violin plots represent the median (white dot), thick lines indicate 25th/75th percentiles, and the thin line extends until the most extreme data points not considered outliers.

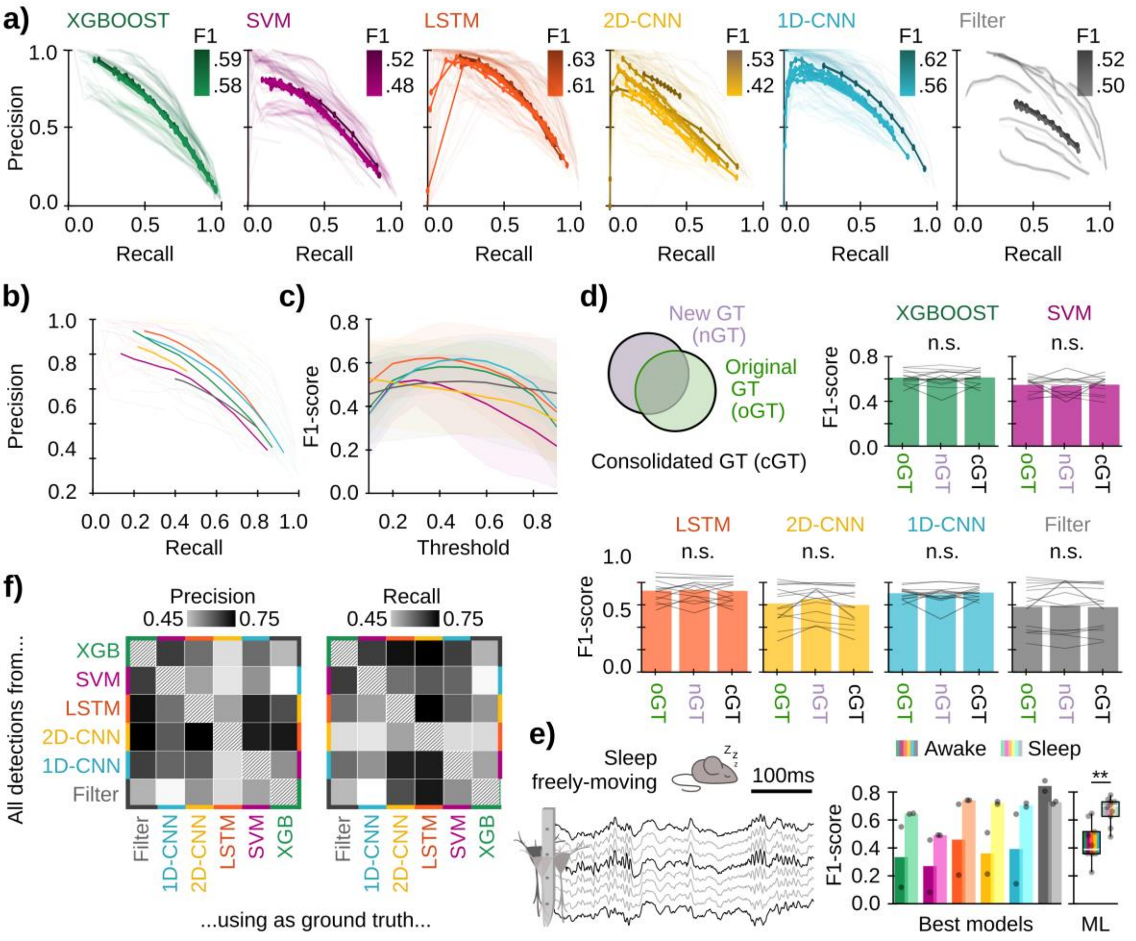

Supplementary Figure 4. Precision-Recall curves of optimized models. a) Precision (P) vs Recall (R) curves for the 10-best models of each architecture, and the 10-best filters of all possible parametric combination. Each dot represents P-R values for a particular threshold. Each line represents the performance of one trained model, colored by its maximal F1 (mean of all sessions is plotted in dark color; sessions are light colored). **b)** P-R curves for the best model of each architecture (all thresholds). Thick lines represent mean values. Thin lines curves are individual test sessions. **c)** F1-score as a function of the threshold. Data reported as mean±95% confidence interval for test sessions. **d)** Performance against other manually tagged ground truths. Three different ground truths were used: the original ground truth (oGT), annotated by the expert whose labels were used to trained the ML models; a new ground truth (nGT), annotated by a different expert; and the consolidated ground truth (cGT), computed as oGT U nGT. Kruskal-Wallis, non-significant for all cases; post hoc tests. **e)** Performance of the best model of each architecture and the best filter, during freely-moving awake (dark colors) and sleep (light colors). Signals from linear arrays were sampled around the CA1 cell body layer and expanded by interpolation to meet the 8-channel input of the ML models. Boxplots show the median (white line), percentile 25% and 75% (box size), and SD (error bars). Kruskal-Wallis over ML models, Chi(4)=10.03, p=0.0015. Post hoc tests: **, p<0.01. **f)** Similarity between the events predicted by the best model (maximum F1) of each architecture and filter. Models shown are the ones with maximum F1. To measure the similarity, we computed the mean Precision (right) and Recall (left) across test sessions have been computed, and used detected SWR events of models in the y-axis as detections, and detected events of models in the x-axis as ground truth.

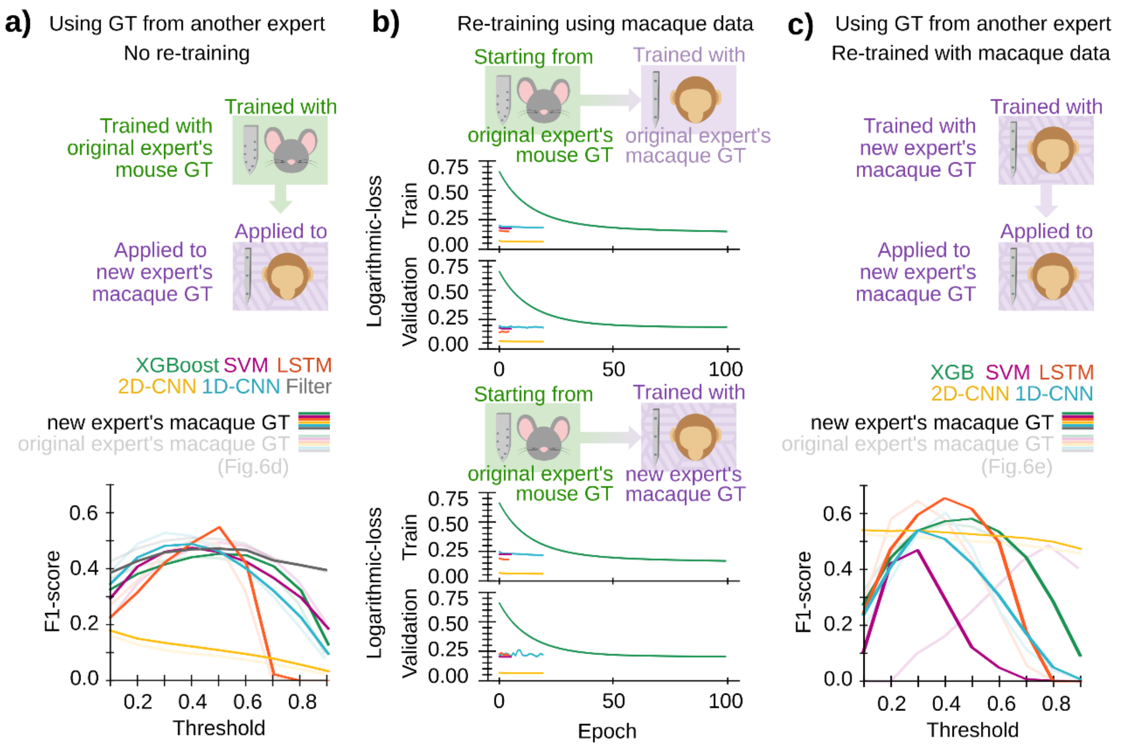

Supplementary Figure 5. Evaluating effects of the expert's ground-truth in detection of monkey SWR. a) The best model of each architecture trained in mouse data was applied to detect SWRs on the macaque data. We evaluated the F1-score with the ground truth of a new expert in the macaque dataset, instead of the original expert's mouse ground truth (Fig.6d, copied here in light colors). Note how some models perform better with the new expert's GT (e.g., LSTM) while others worsen (e.g. SVM). **b)** Learning curves of the re-trained models to see the effectiveness of the transfer/learning. Top: re-training using original expert's macaque data for both train and validation datasets. Bottom: same, but using the new expert's macaque data. Note that only XGBoost did not transfer efficiently to the new dataset. **c)** Results of model re-training using new expert's macaque ground truth to both re-train and evaluate, instead of the original expert's macaque ground truth (Fig.6e, copied here in light colors)

Supplementary Table 1. Sessions and animals used for the different analysis. Summary of the experimental sessions used for training, validation, test and generalization tests.

Animal	Session	Duration (s)	# SWRs
Training			
Amigo2_1	hippo_2019-07-11_11-57-07_1150um	2398.86	1309
Som_2	hippo_2019-07-24_12-01-49_1530um	1036.25	485
Validation			
Dlx1	2021-02-12_12-46-54	1021.34	211
Thy7	2020-11-11_16-05-00	744.21	1064
Test			
Calb20	2021-01-22_13-08-20	1203.23	412
Dlx1	2021-02-12_12-24-56	1200.67	254
Thy1GCam1	2020-12-18_13-16-03	708.92	301
Thy1GCam1	2020-12-18_13-32-27	669.15	412
Thy1GCam1	2020-12-18_14-40-16	613.75	245
Thy1GCam1	2020-12-18_14-56-54	725.4	237
Thy1GCam1	2020-12-21_14-58-51	630.61	115
Thy1GCam1	2020-12-21_15-11-32	651.16	159
Thy1GCam1	2020-12-21_15-26-01	682.22	165
Thy7	2020-11-11_16-21-15	763.67	926
Thy7	2020-11-11_16-35-43	701.99	656
Thy9	2021-03-16_12-10-32	1516.65	264
Thy9	2021-03-16_14-31-51	1201.19	274
Thy10	2021-06-01_13-28-27	626.62	318
Thy10	2021-06-15_15-28-56	976.90	566
PV6	2021-04-19_14-02-31	1051.75	422
PV7xChR2	2021-05-18_13-08-23	958.53	80
PV7xChR2	2021-05-18_13-24-33	855.42	121
PV7xChR2	2021-05-18_13-08-23	958.53	88
Generalization (Awake/Sleep & Macaque)			
REMI2 - mouse	2022-02-01	764.00	524
REMI3 - mouse	2022-04-08	922.00	467
FN - monkey	CODY_2022-10-15	6900.00	3967
