# Supplementary material for: A machine learning toolbox for the analysis of sharp-wave ripples reveals common waveform features across species
Source: Commun Biol. 2024 Mar 4;7:211. doi: 10.1038/s42003-024-05871-w (PMC10912113; doi:10.1038/s42003-024-05871-w)
Supplement: Supplementary file 2 — Reporting Summary [file 42003_2024_5871_MOESM2_ESM.pdf]

nature portfolio

Corresponding author(s): Liset M de la Prida

Last updated by author(s): Jan 16, 2024

Reporting Summary

Nature Portfolio wishes to improve the reproducibility of the work that we publish. This form provides structure for consistency and transparency in reporting. For further information on Nature Portfolio policies, see our Editorial Policies and the Editorial Policy Checklist.

Statistics

For all statistical analyses, confirm that the following items are present in the figure legend, table legend, main text, or Methods section.

n/a	Confirmed	
☐	☒	The exact sample size (n) for each experimental group/condition, given as a discrete number and unit of measurement
☐	☒	A statement on whether measurements were taken from distinct samples or whether the same sample was measured repeatedly
☐	☒	The statistical test(s) used AND whether they are one- or two-sided<br>*Only common tests should be described solely by name; describe more complex techniques in the Methods section.*
☐	☒	A description of all covariates tested
☐	☒	A description of any assumptions or corrections, such as tests of normality and adjustment for multiple comparisons
☐	☒	A full description of the statistical parameters including central tendency (e.g. means) or other basic estimates (e.g. regression coefficient) AND variation (e.g. standard deviation) or associated estimates of uncertainty (e.g. confidence intervals)
☐	☒	For null hypothesis testing, the test statistic (e.g. F, t, r) with confidence intervals, effect sizes, degrees of freedom and P value noted<br>*Give P values as exact values whenever suitable.*
☒	☐	For Bayesian analysis, information on the choice of priors and Markov chain Monte Carlo settings
☐	☒	For hierarchical and complex designs, identification of the appropriate level for tests and full reporting of outcomes
☐	☒	Estimates of effect sizes (e.g. Cohen's d, Pearson's r), indicating how they were calculated

Our web collection on statistics for biologists contains articles on many of the points above.

Software and code

Policy information about availability of computer code

Data collection	Open ephys
Data analysis	Python (3.9), Matlab 2019b. Python packages numpy, tensorflow, keras, xgboost, scikit-learn, imblearn, matplotlib, pandas, scipy were used. Our models are available at github: https://github.com/PridaLab/rippl-AI.

For manuscripts utilizing custom algorithms or software that are central to the research but not yet described in published literature, software must be made available to editors and reviewers. We strongly encourage code deposition in a community repository (e.g. GitHub). See the Nature Portfolio guidelines for submitting code & software for further information.

Data

Policy information about availability of data

All manuscripts must include a data availability statement. This statement should provide the following information, where applicable:

- Accession codes, unique identifiers, or web links for publicly available datasets
- A description of any restrictions on data availability
- For clinical datasets or third party data, please ensure that the statement adheres to our policy

Experimental data supporting the findings of this study are available at: https://figshare.com/projects/cnn-ripple-data/117897

Research involving human participants, their data, or biological material

Policy information about studies with human participants or human data. See also policy information about sex, gender (identity/presentation), and sexual orientation and race, ethnicity and racism.

Reporting on sex and gender	n/a
Reporting on race, ethnicity, or other socially relevant groupings	n/a
Population characteristics	n/a
Recruitment	n/a
Ethics oversight	n/a

Note that full information on the approval of the study protocol must also be provided in the manuscript.

Field-specific reporting

Please select the one below that is the best fit for your research. If you are not sure, read the appropriate sections before making your selection.

☒ Life sciences ☐ Behavioural & social sciences ☐ Ecological, evolutionary & environmental sciences

For a reference copy of the document with all sections, see nature.com/documents/nr-reporting-summary-flat.pdf

Life sciences study design

All studies must disclose on these points even when the disclosure is negative.

Sample size	No statistical method was used to predetermine sample sizes. Electrophysiological mice recordings training set: 1794 events, two sessions from 2 mice; test set: 1275 events; two sessions from 2 mice; validation set: 7586 events; 21 sessions from 8 mice. Data published in Navas-Olive et al., eLife 2020. Electrophysiological macaque recordings: 4133 events from 1 session from 1 macaque.
Data exclusions	Recording intervals with prolonged noise data affecting all channels was not considered for analysis.
Replication	The exact number of replications for each experiment is detailed in text and figures. Detection for all methods was replicated in the validation set (7586 events; 21 sessions from 8 mice) and in the generalization set (4133 events from 1 session from 1 macaque).
Randomization	n/a
Blinding	Ressearchers were blinded during manual ripple annotation.

Reporting for specific materials, systems and methods

We require information from authors about some types of materials, experimental systems and methods used in many studies. Here, indicate whether each material, system or method listed is relevant to your study. If you are not sure if a list item applies to your research, read the appropriate section before selecting a response.

Materials & experimental systems

n/a	Involved in the study	
☒	☐	Antibodies
☒	☐	Eukaryotic cell lines
☒	☐	Palaeontology and archaeology
☐	☒	Animals and other organisms
☒	☐	Clinical data
☒	☐	Dual use research of concern
☒	☐	Plants

Methods

n/a	Involved in the study	
☒	☐	ChIP-seq
☒	☐	Flow cytometry
☒	☐	MRI-based neuroimaging

Animals and other research organisms

Policy information about studies involving animals; ARRIVE guidelines recommended for reporting animal research, and Sex and Gender in Research

Laboratory animals	Data from Mus Musculus and Macaca Mulatta
Wild animals	This study does not involve wild animals
Reporting on sex	Data from both males and females mice were used. A female Macaca Mulatta was used
Field-collected samples	No field collected samples were used in this study.
Ethics oversight	Mouse data used in this study was reported in Navas-ollve et al. eLife 2020 and Sabastian et al., Nat Neu 2023. These data was obtained according to protocols and procedures that follow the Spanish legislation (R.D. 1201/2005 and L.32/2007) and the European Communities Council Directive 2003 (2003/65/CE) for animal research. Monkey data was obtained as in Abbaspoor et al., eLife 2023, with procedures conducted in accordance with the approved protocols and authorized procedures under the local animal care authorities (Institutional Animal Care and Use Committee).

Note that full information on the approval of the study protocol must also be provided in the manuscript.
